# Bon appétit, your phagocyte

**DOI:** 10.1038/s41420-026-03099-7

**Published:** 2026-04-14

**Authors:** Dilara C. Ozkocak, Jascinta P. Santavanond, Maria C. Tanzer, Ivan K. H. Poon

**Affiliations:** 1 Walter and Eliza Hall Institute of Medical Research, Parkville, VIC Australia; 2 Department of Medical Biology, University of Melbourne, Parkville, VIC Australia; 3 Department of Biochemistry and Chemistry, Research Centre for Extracellular Vesicles, La Trobe Institute for Molecular Science, La Trobe University, Melbourne, VIC Australia

**Keywords:** Apoptosis, Cell death and immune response

## Abstract

Every day, billions of cells in the human body undergo apoptosis as part of normal tissue turnover. The swift clearance of these dying cells by phagocytes is an essential process to limit the release of intracellular contents that can disrupt tissue homeostasis and promote inflammation, autoimmunity, and chronic disease. Thus, as with the unseen yet continuous work of a well-run kitchen, effective apoptotic cell clearance sustains multicellular organisms. In cooking, ingredients must be deliberately gathered, trimmed, seasoned, cooked, and plated before becoming part of a satisfying meal. Analogously, apoptotic cells undergo an equally deliberate ‘meal preparation’ process in which they transform themselves to be optimally suited for consumption by professional and non-professional phagocytes. This preparation involves a coordinated suite of modifications, including the secretion of immunomodulatory factors, the internal partitioning of organelles, and the exposure of ‘eat-me’ signals. Additionally, maintaining membrane integrity during apoptosis can be viewed as a ‘protective wrapping’ that preserves ‘edible’ cargo while preventing the inflammatory spillover that would result from premature plasma membrane rupture (PMR). In this perspective, we highlight how these distinct, yet interconnected layers of apoptotic cell ‘cooking’ converge to shape and influence the eventual phagocytic encounter. A deeper understanding of how apoptotic cells prepare themselves for clearance not only reframes our view of cell death, but also offers opportunities to harness or correct these processes in pathological settings where clearance fails.

## Introduction

Timely removal of apoptotic cells, a process known as efferocytosis, is essential to maintain tissue homeostasis as uncleared apoptotic cells can interfere with normal tissue function and elicit unwanted inflammation through the release of danger-associated molecular patterns (DAMPs) following membrane permeabilisation [1–3]. As cellular turnover via apoptosis is continuous throughout life, efferocytosis is important in many contexts such as embryonic [4] and immune development [5], retinal cell maintenance [6] and epithelial cell proliferation [7]. Conversely, defects in efferocytosis can lead to the accumulation of DAMPs, which underpins the aetiology of inflammatory and autoimmune pathologies such as atherosclerosis [8], rheumatoid arthritis [9], inflammatory bowel disease [10], systemic lupus erythematosus [11] and Sjögren’s syndrome [12].

**Fig. 1:**
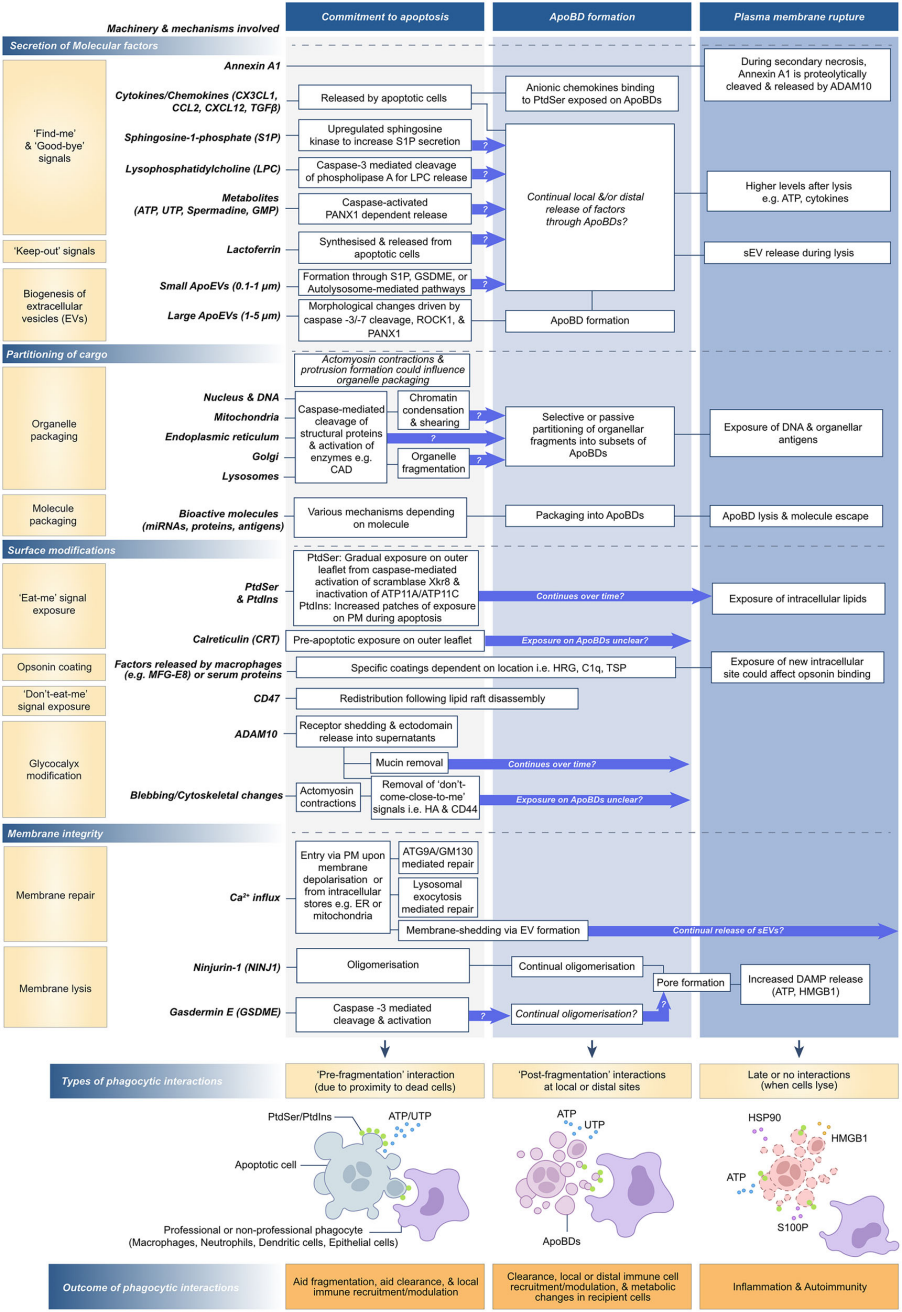
Concurrent events during key stages of apoptosis that ensure appropriate phagocyte interactions. Following apoptosis induction, cells will proceed through distinct stages described herein. The first stage is the commitment to apoptosis (first column), marked by activation of executioner caspases 3 and 7. The following stage (middle column) is where the cell undergoes apoptotic cell disassembly to release membrane-bound large extracellular vesicles (EVs) called apoptotic bodies (ApoBDs). The final stage of the apoptotic programming is where the cell undergoes plasma membrane rupture (final/right column) to leak intracellular contents such as danger-associated molecular patterns (DAMPs). During each stage, cells will initiate parallel events (left panels) including the release of molecular factors, begin the partitioning of intracellular cargo, inititate surface modifications and begin changes to overall cellular membrane integrity. Molecular factors and signals such as annexin-A1 [16], cytokines/chemokines [30, 53], sphingosine-1-phosphate (S1P) [77], lysophosphatidylcholine (LPC) [78], metabolites [26, 53] and lactoferrin [25] will be released following the commitment to apoptosis. Accompanied by this is the simultaneous release of small and large apoptotic EVs through various mechanisms that can span across the different stages of apoptosis [21, 45, 53, 79–82]. Concurrently, apoptotic cells will also induce modifications to the outer plasma membrane, namely through the exposure of phosphatidylserine (PtdSer) and phosphoinositides (PtdIns) [14], calreticulin (CRT) [52, 83], binding of macrophage-specific factors [84], as well as glycocalyx modifications including the redistribution of CD47 [54], receptor shedding through ADAM10 action [16, 57], and the removal of mucins [55, 85]. The partitioning of bioactive cargo within apoptotic cells and ApoBDs can occur after caspase-mediated cleavage of structural proteins and other enzymes [35, 37, 38, 86, 87]. Continual exposure of cargo with organellar origins (like the nucleus/DNA and Golgi) can lead to auto-antigen production. Simultaneously, apoptotic cells will continually exhibit changes to membrane integrity due to Ca^2+^ influx, ninjurin-1 (NINJ1) and Gasdermin E (GSDME) activation [60, 63, 65, 79, 88]. It should be noted that in certain settings, cells do not need to undergo fragmentation to enter the later stages of plasma membrane rupture. Importantly, each event can occur concurrently and, where certain events influence or continue into different stages that require further exploration, is denoted by blue arrows. Taken together, all events and stages will influence the timing and types of phagocytic interactions occurring (bottom yellow boxes), including pre-fragmentation to further initiate fragmentation, post-fragmentation efferocytosis to ensure removal of dying cell debris and lastly, ‘late’ interactions marked by widespread DAMP release, which may or may not recruit phagocytes. Ultimately, the timing of efferocytotic mechanisms will influence the outcome on physiological systems (orange boxes) to maintain homoeostasis or contribute to inflammation.

To achieve rapid removal of apoptotic cells, commitment to apoptotic cell death will trigger important downstream molecular processes that ensure appropriate communication between dying cells and phagocytes, such as macrophages and epithelial cells [13]. Similar to cooking at Michelin-starred restaurants, apoptotic cells (i.e. the ‘meal’) will be carefully prepared for phagocytes to eat. For example, the gradual exposure of the phospholipid phosphatidylserine (PtdSer) on the surface of apoptotic cells was identified as a hallmark of apoptosis [14] and functions as a key ‘eat-me’ signal to help phagocytes to distinguish apoptotic cells from live cells [15]. Concomitantly, apoptotic cells can secrete soluble factors called ‘find-me’ signals to attract phagocytes towards the site of cell death [8, 16–20], as well as undergo cell fragmentation to generate petite ‘bite-sized’ pieces for phagocytes to engulf efficiently [21]. It is worth noting that depending on the physiological context, distinct cell type(s) will undergo apoptosis and be cleared by the corresponding phagocyte(s) at that location. Importantly, this specific ‘pairing’ of apoptotic cells with phagocytes can dictate downstream responses [22, 23]. Furthermore, additional factors in the cell death environment, such as the presence of infectious agents and/or other forms of cell death (e.g. lytic cell death), will also influence the cellular responses following cell clearance [23]. Thus, it is important to acknowledge that the apoptotic ‘meal’ and the properties of phagocytes will change accordingly under different physiological and pathological settings; however, this complexity will not be addressed in detail herein. In this perspective, we examine the complex, concurrent events that unfold during apoptosis and how they collectively prepare the ‘meal’ for phagocytes. By clarifying when and how this meal is prepared and subsequently consumed, we illustrate how these stages shape distinct downstream responses. To define the ‘meal preparation’ phase of efferocytosis, the progression of apoptosis will be divided into 3 key stages: (i) commitment to apoptosis, (ii) cell fragmentation, and (iii) membrane lysis (Fig. 1). Depending on the stage of meal preparation, this will influence the soluble factors that are released, surface modifications available for phagocytes to engage with, which phagocytes could be interacting with apoptotic materials, and lastly, the cargo within apoptotic materials.

## Secretion of molecular factors

Following the commitment to apoptosis, apoptotic cells can release a range of molecular factors collectively termed as ‘find-me’ signals to capture the attention of neighbouring phagocytes [16, 18–20]. These molecular factors come in a variety of ‘scents’ to lure the phagocytes to their meal/site of cell death and to prime their appetite for downstream processes. During the early stages of apoptosis, the opening of pannexin 1 (PANX1) channels following caspase 3/7-mediated cleavage allows for the release of nucleotides such as adenosine triphosphate (ATP) and uridine triphosphate (UTP), which can function as chemotactic factors and are sensed by purinergic receptor P_2_Y_2_ expressed on macrophages[18, 19]. Lipid molecules such as S1P and LPC can also be released by apoptotic cells through caspase 3 mediated activation of the calcium-independent phospholipase A_2_ and act as ‘find-me’ signals [24]. It is important to note that whilst the release of ATP at lower levels promotes an anti-inflammatory response by initiating efferocytosis, the lysis of apoptotic material (discussed below) could lead to high levels of extracellular ATP, which could act as DAMP to trigger an inflammatory immune response[1].

To establish and maintain an anti-inflammatory environment, apoptotic cells can also release ‘keep-out’ and ‘good-bye’ signals. Lactoferrin (a ‘keep-out’ signal) can be released by apoptotic cells and limit inflammation by preventing the recruitment of neutrophils to the site of cell death [25]. Metabolites including spermidine and guanosine 5’‑monophosphate (GMP) (‘good‑bye’ signals) are released through caspase-activated PANX1 channels and can alter the gene expression of neighbouring immune cells to initiate a wound healing and anti‑inflammatory response [26].

In addition to the release of soluble factors, apoptotic cells can form a heterogeneous population of apoptotic cell-derived extracellular vesicles (ApoEVs). Small ApoEVs ( ~ 0.1–1 μm in diameter) are described to be formed through S1P, GSDME, or autolysosome-mediated pathways [27, 28]. Large ApoEVs, known as apoptotic bodies (ApoBDs), are typically ~1–5 μm in diameter and are formed through the morphological changes of apoptosis, regulated by caspase 3/7 cleavage of rho-associated kinase 1 (ROCK1) and PANX1 [21, 29]. Notably, apoptotic cell-derived chemokines such as CX_3_CL1 and CCL12 have been shown to be associated with or bind to exposed PtdSer on ApoEVs, to promote the migration of phagocytes towards apoptotic cells [20, 30]. This suggests that ApoEVs can serve as messengers, carrying the ‘scent’ to phagocytes to initiate the efferocytotic response. However, whether other ‘find-me’, ‘good-bye’ or ‘keep-out’ signals as mentioned above can be carried within or on the surface of ApoEVs to mediate continual release or effect at distal sites is yet to be fully investigated.

## Partitioning of cargo

The fragmentation and packaging of cellular material is of high importance in determining the ‘flavour’ and ‘nutritional content’ of an apoptotic meal. It is key in facilitating functional changes in phagocytes, namely by powering continual rounds of efferocytosis [31], metabolic changes [32] and phenotypic reprogramming [33] (Fig. 1).

Following the initiation of apoptosis, the ‘ingredients’ of an apoptotic meal are trimmed and prepared by the mobilisation and the action of caspases to initiate cellular breakdown. Executioner caspases 3, 6, and 7 proteolytically activate degradative enzymes to initiate the breakdown of nuclear material within apoptotic cells, such as DNA and nuclear lamins A and B [34]. Morphological changes such as membrane blebbing and nuclear condensation are also proposed to play key roles in shearing nuclear material [35]. For organelles such as the mitochondria, the recruitment of apoptosis regulator and pore-forming protein BAX to cardiolipin-rich areas of the mitochondrial membrane allows the release of cytochrome C, and subsequent fragmentation of the mitochondria in a DRP1-dependent manner [36]. Moreover, the structural components of cells are also degraded, particularly the cytoskeletal network of F-actin, and membranous organelles such as the endoplasmic reticulum [35, 37, 38], and Golgi, through cleavage of giantin, GRASP65 [39], golgin-160, GM130, and p115 [40, 41] to further facilitate cellular breakdown. Despite this, the exact mechanisms underpinning the packaging of various cellular contents into ApoEVs is scarce. Nevertheless, ER-derived and mitochondrial material can be shuttled into ApoEVs during monocyte fragmentation [29]. Bioactive molecules such as miRNA, DNA, and proteins can also be packaged into ApoEVs [27, 29, 42, 43]. In the context of viral infections, viral proteins, including influenza A virus nucleoprotein and SARS-CoV-2 spike protein, as well as infectious virions, can be transferred via ApoBDs [44, 45]. Similar to food poisoning, this transfer of virions through the apoptotic meal can consequently promote viral dissemination as observed for influenza A virus [44], SARS-CoV-2 [45], and norovirus [46]. Whether the shuttling of material into ApoEVs occurs due to chaperone molecules or due to the extensive network of contact-sites between organelles [47] remains to be elucidated.

## Surface modifications

To enable phagocytes to accurately identify apoptotic cells among viable cells, the surface of the apoptotic cells undergoes various molecular changes. As described earlier, the exposure of PtdSer on the surface of apoptotic cells function as one of the most important ‘eat-me’ signal to enable phagocytes to detect and trigger subsequent uptake of apoptotic materials [48]. The gradual exposure of PtdSer during apoptosis is elegantly controlled by caspase-mediated activation of scramblase and inactivation of flippase (reviewed in detail by *Nagata* et al. *2020* [49]). Besides PtdSer, exposure of other phospholipids, including phosphoinositides, can also function as ‘eat-me’ signals [50]. Under certain conditions, such as ER stress, apoptotic cells can expose additional ‘eat-me’ signals, including calreticulin, to aid cell clearance [51, 52]. Importantly, different phagocytic subsets are equipped with a distinct repertoire of receptors (e.g. TIM-1/-4, BAI1, Stabilins, CD14) that can directly identify these surface modifications (reviewed in detail by *Moon* et al. *2023* [2]). Furthermore, depending on the tissue context, apoptotic cells can also be ‘decorated’ or ‘glazed’ with a range of opsonins such as MFG-E8, Protein S, C1q and TSP, which act as bridging molecules to receptors such as Integrin αVβ3 and TAM receptors on phagocytes to trigger subsequent clearance. It should be noted that these opsonins can be released by phagocytes themselves or present in the serum/extracellular milieu in steady state [53]. Depending on the tissue context, as ApoEVs can be trafficked away from the site of cell death, ApoEVs could be decorated with a complex ‘corona’ to regulate downstream processes.

Besides exposing additional signals on the apoptotic surface to help phagocytes to identify the ‘meal’, it is equally important to remove the molecular ‘glad wrap’ that is normally present on healthy cells. Engagement of CD47 and CD31 on healthy cells with SIRPα and CD31 on phagocytes prevents accidental removal of healthy cells [8, 54]. Also known as ‘don’t eat me’ signals, CD47 and CD31 are redistributed or modified during apoptosis to enable apoptotic cell recognition and uptake. More recently, exposure of ‘eat-me’ signals away from the exofacial glycocalyx (e.g. HA, CD44) on apoptotic cells through the formation of membrane blebs[55] and/or ADAM10-mediated shedding of mucins [56, 57] has been shown to aid phagocyte recognition and uptake. Removal of these barriers is important for the efferocytic synapse to form appropriately to trigger recognition and engulfment, possibly by removing the physical constraint required for ‘eat-me’ signal-receptor interaction [58]. Ultimately, the combination of signals exposed on apoptotic materials also contributes to the ‘flavour’ of the meal.

## Maintenance of membrane integrity

The plasma membrane (PM) undergoes various structural and morphological changes that compromise its integrity during the progression of cell death. Primarily, potassium (K^+^) efflux and calcium (Ca^2+^) influx caused by membrane perturbations can continue throughout apoptosis prior to the onset of secondary necrosis, a post-apoptotic state characterised by excess release and passive leakage of intracellular contents such as DAMPs [1]. The release of DAMPs and subsequent receptor activation is linked to the pathology of various autoimmune, inflammatory, and neurodegenerative diseases [1, 3]. From a culinary perspective, this stage can be thought of as the apoptotic meal becoming ‘overcooked’ - where the PM’s protective casing is damaged, the packaged cargo spills out in an uncontrolled manner, transforming an initially ‘neatly prepared’ and immunologically silent meal into a ‘messy’ and inflammatory one. Thus, ensuring the coordination between membrane repair and membrane lysis is a vital aspect in determining the downstream consequences of apoptosis.

PMR is primarily initiated through the action of specialised protein executioners. Pore-forming proteins such as members of the GSDM family and NINJ1 promote PMR across multiple modes of cell death, including apoptosis, pyroptosis, and, more recently, ferroptosis [59–62]. Through their pore-forming activity, GSDMD, GSDME, and NINJ1 facilitate leakage of intracellular DAMPs such as ATP and HMGB1, as well as cytokines that engage immune receptors and shape downstream responses [1, 62]. Importantly, the timing at which permeabilisation occurs can directly influence apoptotic cell fragmentation into ApoBDs, whereby early permeabilisation can limit ApoBD formation[28]. Thus, controlled and delayed membrane rupture permits the orderly packaging of cellular contents into ApoBDs. One mechanism that prevents premature membrane lysis despite activation of pro-lytic factors is the concurrent repair of the PM. Healthy cells deploy multiple repair strategies triggered by Ca^2+^ influx, including inward or outward budding, donation of membrane from intracellular compartments, and removal of damaged regions by neighbouring cells [63]. Under conditions of mechanical stress or damage, additional membrane sources such as lysosomes can also be mobilised through lysosomal exocytosis [64]. ATG9A, a lipid scramblase involved in autophagosome formation, cooperates with IQGAP1 and ESCRT proteins across diverse cell death contexts to protect against PMR [65]. Whether other organelles including the ER, Golgi, and lysosomes, all known reservoirs of lipids for PM homoeostasis [64, 66] can serve as membrane ‘donors’ during apoptotic PM repair remains to be clarified. When membrane lysis ultimately proceeds, the exposure of intracellular structures such as F-actin can serve as additional damage-associated cues, for instance, Clec9A on dendritic cells recognises exposed F-actin, thereby promoting cross-presentation and altering the nature of the phagocytic responses [67]. Thus, the balance between repair and lysis not only dictates the structural fate of dying cells but also the immunological information conveyed to phagocytes.

## Types of phagocytic interactions and associated outcomes

Ultimately, just as food needs to be eaten before it spoils and makes us sick, dead cell material must be cleared before it causes harm. In healthy tissues, this clearance is remarkably efficient - so much so that dead cells are rarely detectable under homeostatic conditions [68]. In vitro, phagocytes can engulf apoptotic cells within minutes, and in vivo, injected apoptotic cells disappear in a short timeframe [69, 70]. This rapid disposal process underpins the physiological importance of timely efferocytosis. As mentioned above, PtdSer exposure is a key ‘eat-me’ signal that likely drives this speed and efficiency, meanwhile, other aspects of ‘meal preparation’, remain less well understood. Nevertheless, ApoEVs have been identified as important sentinels that may travel farther through tissues and be readily engulfed. Although currently undefined, there is a potential for ApoEVs to power continual rounds of efferocytosis and even prime macrophages for subsequent larger meals [27]. In germinal centres, ApoBDs can promote the maturation of follicular macrophages into tingible body macrophages, specialised for clearing debris in lymph nodes [5]. In certain pathological settings, however, eating too much, or ‘unhealthy’ foods, can impact clearance. For example, in atherosclerosis, lipid-engorged macrophages (foam cells) may lose their clearance capacity, thereby exacerbating disease progression [71, 72].

Additionally, not all meals are equal. The macrophage response depends not only on whether cells die, but on the cell type and death modality. Apoptotic neutrophils uniquely trigger a wound-healing programme in macrophages, while other cell types or death modes fail to do so [23]. Furthermore, dead cells containing pathogens [44] or exposure to H_2_O_2_ [73] can drive efferocytosis toward inflammatory outcomes, rather than tolerance. Notably, when consumption is delayed, even a well-prepared apoptotic meal can spoil, leading to the accumulation and secretion of secondary necrotic materials. Although necrotic materials can be efficiently engulfed [74, 75], they may reprogramme macrophages in distinct ways.

Local ‘diners’ also matter. Immune responses to dead cells vary across tissues, with phagocytes such as Kupffer cells in the liver and microglia in the brain [76]. These differences likely reflect variation in the expression of PtdSer and DAMP receptors, local immune context, and the need for antigen presentation. However, in disease, phagocytic systems may be overwhelmed, defective, or altered by the type of dying cell or tissue environment. The signals exposed, cargo content, and local context altogether shape whether engulfment maintains homoeostasis or fuels inflammation. More in vivo studies are needed to resolve how these different ‘meals’ could reprogramme ‘diners’ such as macrophages and other phagocytes and subsequently influence tissue homoeostasis and disease progression.

Finally, the timing of phagocyte engagement with apoptotic cells is a critical determinant of clearance outcomes. When phagocytes encounter apoptotic cells within the optimal window (when ‘eat-me’ signals are displayed, the glycocalyx is remodelled, membrane integrity is maintained, and cellular contents are appropriately packaged into ApoBDs), they initiate programmes that support tissue repair, resolution of inflammation, and proliferative cues for neighbouring cells [2, 8, 53]. However, as mentioned above, if phagocyte interaction is delayed and apoptotic cells progress toward secondary necrosis, the resulting shift in molecular signals can redirect phagocytes toward inflammatory responses [1, 3, 22]. Distinct phagocyte subsets may differentially interpret these late-stage cues, further diversifying downstream outcomes.

## Conclusion

Across the three major stages of apoptosis: commitment to cell death, cell fragmentation, and membrane lysis, apoptotic cells undergo a series of controlled changes that remodel their surface, release chemoattractants, and package or expose intracellular cargo in ways that guide phagocyte engagement. The timing of phagocyte interaction is therefore critical: early and appropriately matched encounters foster tissue repair, resolution of inflammation, and maintenance of homoeostasis, whereas delayed or mismatched interactions may redirect phagocytes toward inflammatory outcomes. Although the specific apoptotic cues and phagocyte responses vary across tissues and disease states, the principle remains the same: the quality and timing of the apoptotic meal, together with the identity and readiness of the diner, collectively determine the immunological consequences of efferocytosis. Understanding these dynamics offers new opportunities to understand cell clearance in pathological settings.
